# SARS-CoV-2 spike protein diversity at an intra-host level, among SARS-CoV-2 infected individuals in South Africa, 2020 to 2022

**DOI:** 10.1371/journal.pone.0286373

**Published:** 2023-05-30

**Authors:** Kathleen Subramoney, Nkhensani Mtileni, Ashlyn Davis, Jennifer Giandhari, Houriiyah Tegally, Eduan Wilkinson, Yeshnee Naidoo, Yajna Ramphal, Sureshnee Pillay, Upasana Ramphal, Andiswa Simane, Bhaveshan Reddy, Bonolo Mashishi, Nonhlanhla Mbenenge, Tulio de Oliveira, Burtram C. Fielding, Florette K. Treurnicht

**Affiliations:** 1 School of Pathology, Faculty of Health Sciences, University of the Witwatersrand, Johannesburg, South Africa; 2 Department of Virology, National Health Laboratory Service, Charlotte Maxeke Johannesburg Academic Hospital, Johannesburg, South Africa; 3 KwaZulu-Natal Research Innovation and Sequencing Platform (KRISP), Nelson R Mandela School of Medicine, University of KwaZulu-Natal, Durban, South Africa; 4 Centre for Epidemic Response and Innovation (CERI), School of Data Science and Computational Thinking, Stellenbosch University, Stellenbosch, South Africa; 5 Molecular Biology and Virology Research Laboratory, Department of Medical BioSciences, University of the Western Cape, Cape Town, South Africa; Emory University, UNITED STATES

## Abstract

Intra-host diversity studies are used to characterise the mutational heterogeneity of SARS-CoV-2 infections in order to understand the impact of virus-host adaptations. This study investigated the frequency and diversity of the spike (S) protein mutations within SARS-CoV-2 infected South African individuals. The study included SARS-CoV-2 respiratory samples, from individuals of all ages, received at the National Health Laboratory Service at Charlotte Maxeke Johannesburg Academic hospital, Gauteng, South Africa, from June 2020 to May 2022. Single nucleotide polymorphism (SNP) assays and whole genome sequencing were performed on a random selection of SARS-CoV-2 positive samples. The allele frequency (AF) was determined using TaqMan Genotyper software for SNP PCR analysis and galaxy.eu for analysis of FASTQ reads from sequencing. The SNP assays identified 5.3% (50/948) of Delta cases with heterogeneity at delY144 (4%; 2/50), E484Q (6%; 3/50), N501Y (2%; 1/50) and P681H (88%; 44/50), however only heterogeneity for E484Q and delY144 were confirmed by sequencing. From sequencing we identified 9% (210/2381) of cases with Beta, Delta, Omicron BA.1, BA.2.15, and BA.4 lineages that had heterogeneity in the S protein. Heterogeneity was primarily identified at positions 19 (1.4%) with T19IR (AF 0.2–0.7), 371 (92.3%) with S371FP (AF 0.1–1.0), and 484 (1.9%) with E484AK (0.2–0.7), E484AQ (AF 0.4–0.5) and E484KQ (AF 0.1–0.4). Mutations at heterozygous amino acid positions 19, 371 and 484 are known antibody escape mutations, however the impact of the combination of multiple substitutions identified at the same position is unknown. Therefore, we hypothesise that intra-host SARS-CoV-2 quasispecies with heterogeneity in the S protein facilitate competitive advantage of variants that can completely/partially evade host’s natural and vaccine-induced immune responses.

## Introduction

The primary function of the coronavirus spike glycoprotein (S protein) is to facilitate virus attachment and infection of host cells [1]. Variations occurring within its amino acid (aa) sequence could influence the interaction with the host receptor, viral pathogenesis, transmissibility and infectivity [1]. Similarly, since the emergence of SARS-CoV-2 in 2019, the S protein has evolved rapidly, resulting in diverse lineages of which some manifested as variants of concern (VOC) [2]. Globally waves of infections were mainly driven by VOCs of the Alpha, Beta, Delta, Gamma, and Omicron lineages. In South Africa, SARS-CoV-2 Wuhan-Hu1 wild-type strain dominated the first coronavirus disease of 2019 (COVID-19) wave, the second and third waves were dominated by Beta and Delta VOCs respectively, and the fourth and slowly emerging fifth waves were dominated by Omicron BA.1, BA.2, BA.4 and BA.5 lineages [3–7]. The continuous emergence of new SARS-CoV-2 lineages overtime and the reversion of mutations together with recombination within the SARS-CoV-2 genome, predominantly in the S protein, could result in strains increasing resistant to antibody-mediated neutralisation. This highlights the need for characterisation of intra-host SARS-CoV-2 S protein diversity.

Several studies have shown that studying intra-host SARS-CoV-2 S protein diversity leads to further insight and understanding of SARS-CoV-2 transmission dynamics and could be important for the improvement of SARS-CoV-2 vaccines [8–11]. The Alpha, Beta, and Delta variants showed escape from neutralising antibodies induced by the dominant vaccine immune epitopes in the S protein. Circulation of these variants also resulted in varying efficacy (50–90%) of licenced SARS-CoV-2 vaccines, which comprised the Wuhan-Hu1 derived S protein. In addition, spatiotemporal analysis of intra-host single nucleotide variants (iSNVs) showed increased genetic diversity following symptomatic infections. In South Africa, iSNVs analysis coupled with bottleneck transmission, was successfully used to determine the transmission dynamics of SARS-CoV-2 in nosocomial outbreaks [11]. The study identified patients with similar iSNV profiles together with the evolution of SARS-CoV-2 infections. A limitation of the study was that intra-host variants were analysed at a nucleotide level with limited focus being placed on the impact of variants on possible immune escape based on aa variations.

Although SARS-CoV-2 variants that dominated the waves from 2020 to 2022 in South Africa have been described, the frequency of heterogeneous infections and heterogeneity within each defined lineage and VOC lineage is not well defined. Therefore, the factors that contribute to the emergence of dominant variants, including factors that could affect variant persistence or eventual replacement is still unclear. Intra-host diversity studies will assist with identifying and characterising the frequency of mutations and possible heterogeneity within the SARS-CoV-2 S protein. This will broaden our understanding of the virus-host adaptations, virulence factors, and pathogenicity and potentially provide information for more effective vaccine design. In this study we aimed to determine the diversity of SARS-CoV-2 S protein among SARS-CoV-2 infected individuals and if heterogeneity has an impact on intra-host adaptations.

## Methods

### Study population

The study included persons of all ages from whom respiratory samples were submitted for SARS-CoV-2 diagnosis to the National Health Laboratory Service (NHLS) at Charlotte Maxeke Johannesburg Academic hospital (CMJAH), Gauteng, South Africa, from June 2020 to May 2022. CMJAH received SARS-CoV-2 samples within the Gauteng province, as well as referrals from the Eastern Cape, Free-State KwaZulu-Natal, and Western Cape provinces of South Africa. This study contributed towards the national surveillance for SARS-CoV-2 in South Africa for which formal patient consent was not required. The study was approved by the University of Witwatersrand Human Research Ethics Committee (M210119) and all participants’ data was anonymised and presented in an aggregated form.

### Study samples and sample size

Respiratory specimens submitted for SARS-CoV-2 diagnosis included nasal and nasopharyngeal swabs, throat swabs and oropharyngeal swabs, sputum, tracheal and lung aspirates, bronchoalveolar aspirates or lavages. Residual samples were stored for further characterisation of the infecting strains. Approximately 50–80 SARS-CoV-2 positive samples were randomly selected per week for genomics surveillance from 2020–2022. We strived to include at least 10 samples from each district from which samples were collected per day, for detection of single nucleotide polymorphisms (SNP) in the S protein.

### SARS-CoV-2 diagnosis

The respiratory samples were extracted using three extraction platforms and compatible extractions kits: the MagNa Pure 96 (Roche Diagnostics, German) with the MagNa Pure 96 small volume DNA and viral NA extraction kit (Roche), the microlab® NIMBUS® (Hamilton, United States) systems with the Viral DNA/RNA 200 C extraction kit (Hamilton), and the KingFisher Flex (Thermo Fisher Scientific, United States) semi-automated platform with the MagMAX viral/pathogen nucleic acid isolation kit (Thermo Fisher), according to the manufacturers’ instructions. Multiplex real-time reverse transcription-based polymerase chain reaction (rRT-PCR) assays were performed on total nucleic acid according to the manufacturers’ instructions: a) Allplex™ 2019-nCoV Assay (Seegene Inc., Korea) was tested with the CFX96 real-time platform (Bio-Rad, California, United States); b) TaqPath COVID-19 assay (Thermo Fisher Scientific) was tested with the QuantStudio^TM^ 5 (Thermo Fisher Scientific) real-time platform; c) the cobas^®^ SARS-CoV-2 assay (Roche Diagnostics) was processed with the cobas^®^ 8800 platform (Roche Diagnostics); and d) the Biofire FilmArray RP2.1 assay (BioFire Diagnostic, United States) was processed with the BioFire Torch (BioFire Diagnostic) platform.

### Genotyping to detect single nucleotide polymorphisms (SNPs) associated with specific amino acid mutations in the S protein

Total nucleic acids were extracted from randomly selected samples using the automated KingFisher Flex purification system with the MagMAX™ Viral/Pathogen II nucleic acid extraction kit (Thermo Fisher Scientific), as per manufacturer’s instructions. The TaqMan SARS-CoV-2 S gene singleplex mutation panels (Thermo Fisher Scientific) were used to identify 11 mutations (T20N, delH69/70, delY144, K417N, L452R, E484K, E484Q, N501Y, D614G, P681H and P681R). Results from this assay allowed us to determine the presence of homogeneous and/ or heterogenous (i.e., mixed mutations associated with more than 1 genotype) virus populations within an individual. Analysis was performed using the QuantStudio 5 design and analysis and TaqMan® Genotyper software’s. Alleles that clustered along the x-axis (allele 1 VIC) were represented as homozygous wild type with AF≥ 0.5, alleles that clustered along the y-axis (allele 2 FAM) represented homozygous mutants with AF≥ 0.5, and alleles that clustered between the x-axis and y-axis represented heterogeneous wild type/mutant.

### Next-generation sequencing of SARS-CoV-2 strains

All samples selected for sequencing where submitted to the KwaZulu-Natal Research Innovation and Sequencing Platform (KRISP) for sequencing or were sequenced in-house at the NHLS, Virology Laboratory. Samples were amplified using the ARTIC V3, V4 and V4.1 protocols [3, 12, 13]. At KRISP, libraries were prepared using the Nextera DNA library preparation kits (Illumina, United States) for sequencing performed on the MiSeq platform (Illumina) and ligation sequencing kits (Oxford Nanopore technologies, Oxford, United Kingdom) for libraries prepared for sequencing with the MinION (Oxford Nanopore technologies, United Kingdom) [5, 14]. For in-house sequencing, libraries were prepared using the Nextera DNA kits (Illumina) and 8pM of pooled libraries were loaded together with 1% PhiX control which were sequenced on the MiSeq platform (Illumina). Genome assembly was done using genome detective (https://www.genomedetective.com/app/typingtool/virus/) by KRISP and Exatype (https://sars-cov-2.exatype.com/) online tools. Consensus sequences were analysed using Nextstrain (https://clades.nextstrain.org) and Pangolin (https://pangolin.cog-uk.io/) online tools for variant assignment.

### Variant mutation analysis and identification of heterogeneous infections

FASTQ reads generated from sequencing were analysed using the COVID-19 workflows with galaxy.eu [15]. To generate allele frequencies for major and minor variants at the aa level across the S protein: for Illumina pair-end reads we ran the “COVID-19: variation analysis on ARTIC PE data (V0.5)” together with the “COVID-19: variation analysis reporting” workflow; and the “COVID-19 variation analysis of ARTIC ONT (V0.3.1)” workflow was used for MinION single reads. The final output from galaxy.eu indicated if the sequence data passed the quality checks (indicated as “PASS”). Only the sequences that passed were included in this analysis. Heterogenous mutations were identified if the allele frequency ranged from 0.1 to 1.0 for more than 1 mutation.

### Data analysis

Descriptive analysis was done across different age groups (<5, 5–14, 15–24, 25–44, 45–60, and >60 years), gender, province, and specimen collection site (individuals that sought community screen and test services, healthcare workers staff clinic, in-patient wards, out-patient wards, mortuary). Intra-host sequences and genotyping data were analysed to determine the proportion of individuals infected with homogeneous and/or heterogeneous variant populations. A box-whisker analysis of heterogeneous aa mutations was used to display the intra-host frequency of heterogeneous SNPs across the S protein. A time-tree was generated by running our sequence dataset through a NextStrain custom build (https://github.com/nextstrain/ncov) and the most prevalent heterogeneous mutation positions were selected for analysis. The positions with heterogeneous mutations were compared to the global prevalence of mutations at the same positions from 2020 through to 2022, using GISAID CoVServer (https://www.gisaid.org/epiflu-applications/covsurver-mutations-app/; [16]).

## Results

### Demographic characteristics of study population

Over the study period a total of 667 269 respiratory samples were tested, of which 107 495 (16.1%) tested positive for SARS-CoV-2. A total of 2982/107 495 (2.8%) SARS-CoV-2 positive samples were analysed to assess the proportion with mixed infections. Of these, 20.3% (601/2982) were analysed by SNP PCR only, 68.2% (2034/2982) were sequenced and 11.6% (347/2982) were both were analysed by SNP PCR and sequenced.

Just under 50% of SARS-CoV-2 positive samples belonged to adults in age group 25–44 years (1351/2982; 45.3%), followed by older adults aged 45–60 years (594/2982; 20.0%) (Table 1). The proportion of females (57.9%; 1726/2982) was greater than males (39.6%; 1181/2982). The majority of samples were from individuals residing in Gauteng province (2830/2982; 95.0%). Individuals that were out-patients and in-patients represented 11.5% (344/2982) and 8.1% (242/2982) respectively, while 77.9% (2324/2982) of individuals were from community screen and testing (CST) (Table 1). ARV clinic and cardiac clinic attendees represented 25.0% (86/344) and 0.6% (2/344) of out-patients respectively.

**Table 1:** Demographics of individuals with SARS-CoV-2 included in this study (N = 2982).

Demographics	Genotyped (n/N, (%))
***Age group (years)***	
*<5*	35/2982 (1.2)
*5–14*	243/2982 (8.1)
*15–24*	444/2982 (14.9)
*25–44*	1358/2982 (45.5)
*45–60*	594/2982 (20.0)
*>60*	220/2982 (7.4)
*Unknown*	86/2982 (2.9)
*** ***	
***Gender***	
*Female*	1726/2982 (57.9)
*Male*	1181/2982 (39.6)
*Unknown*	75/2982 (2.5)
* *	
***Province/ District***	
*Eastern Cape*	147/2982 (4.9)
*Free-State*	1/2982 (0.0)
*Gauteng*	2830/2982 (95.0)
*KwaZulu-Natal*	3/2982 (0.1)
*Western Cape*	1/2982 (0.0)
* *	
***Specimen collection site***	
*Community screen and test*	2324/2982 (77.9)
*Healthcare workers staff clinic*	59/2982 (2.0)
*In-patient ward*	242/2982 (8.1)
*Out-patient ward*	344/2982 (11.5)
*Mortuary*	13/2982 (0.4)

**Fig 1:**
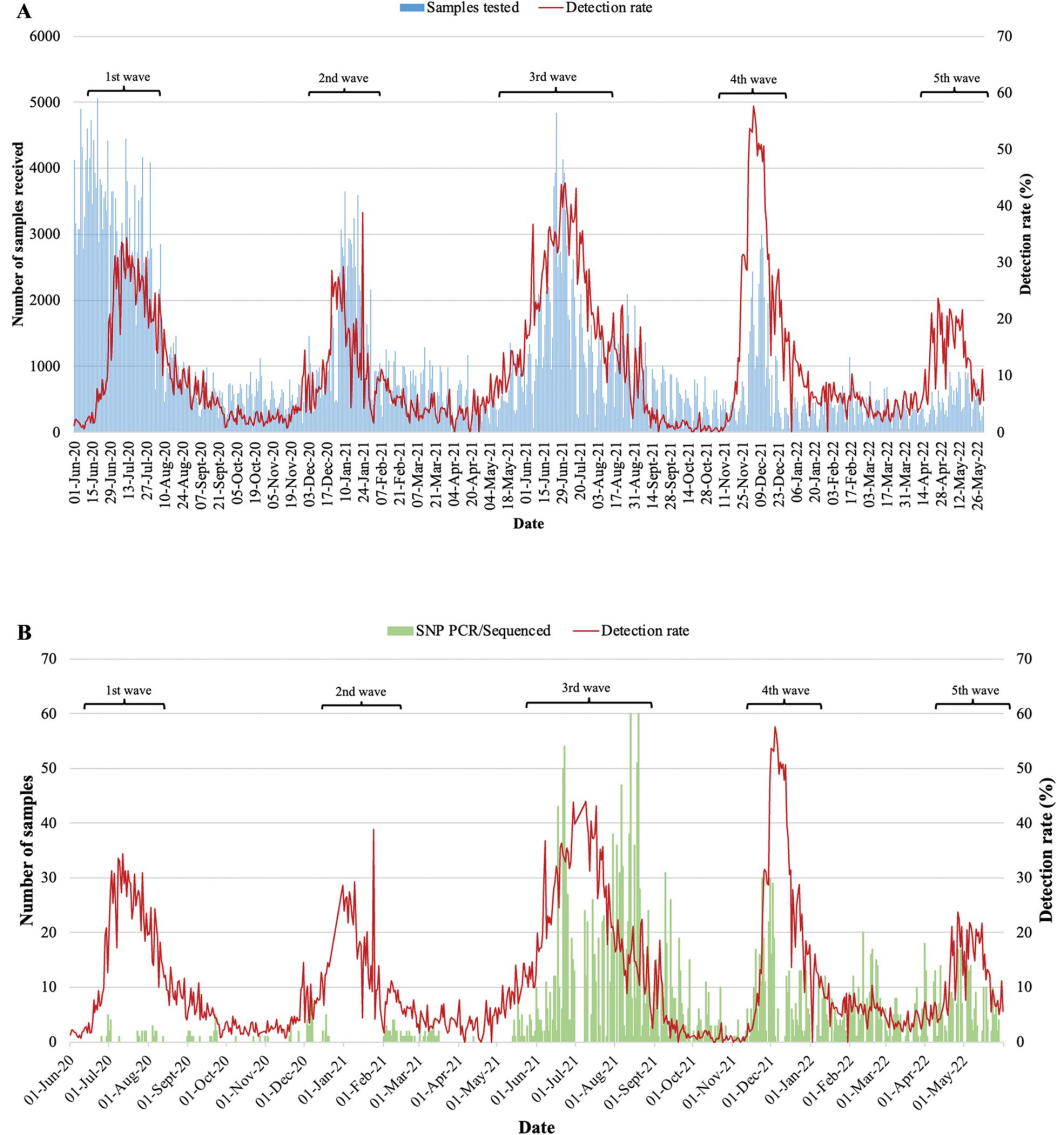
SARS-CoV-2 detection rate among respiratory samples tested from June 2020 to May 2022. A) Blue bars represent the number of samples received for SARS-CoV-2 diagnostics and the red line graph represents the detection rate over time. B) Green bars represent the number of SARS-CoV-2 samples that were tested by SNP PCR and/or sequenced and the red line graph represents the detection rate over time.

Five distinct SARS-CoV-2 waves of infection were observed during the study period from 01 June 2020 through 31 May 2022, which were indicative of the 1st (SARS-CoV-2 Wuhan-lineage dominant), 2^nd^ (Beta dominant), 3^rd^ (Delta dominant), 4^th^ (Omicron BA.1 and BA.2 dominant) and 5^th^ (Omicron BA.4 and BA.5 dominant) SARS-CoV-2 waves of infection (Fig 1A and 1B). The SARS-CoV-2 detection rate ranged from 10.0–34.4% during the 1^st^ wave (28 June to 14 August 2020), 11.0–38.8% in the 2^nd^ wave (14 December 2020 to 24 January 2021), 10.2–44.0% in 3^rd^ wave (31 May to 27 August 2021), 12.1–57.7% during 4^th^ wave (27 November 2021 to 07 January 2022) and in the 5^th^ wave (16 April to 21 May 2022) it ranged from 11.1–23.8%.

### Frequency and characteristics of heterogeneous SNPs across the S protein

**Fig 2:**
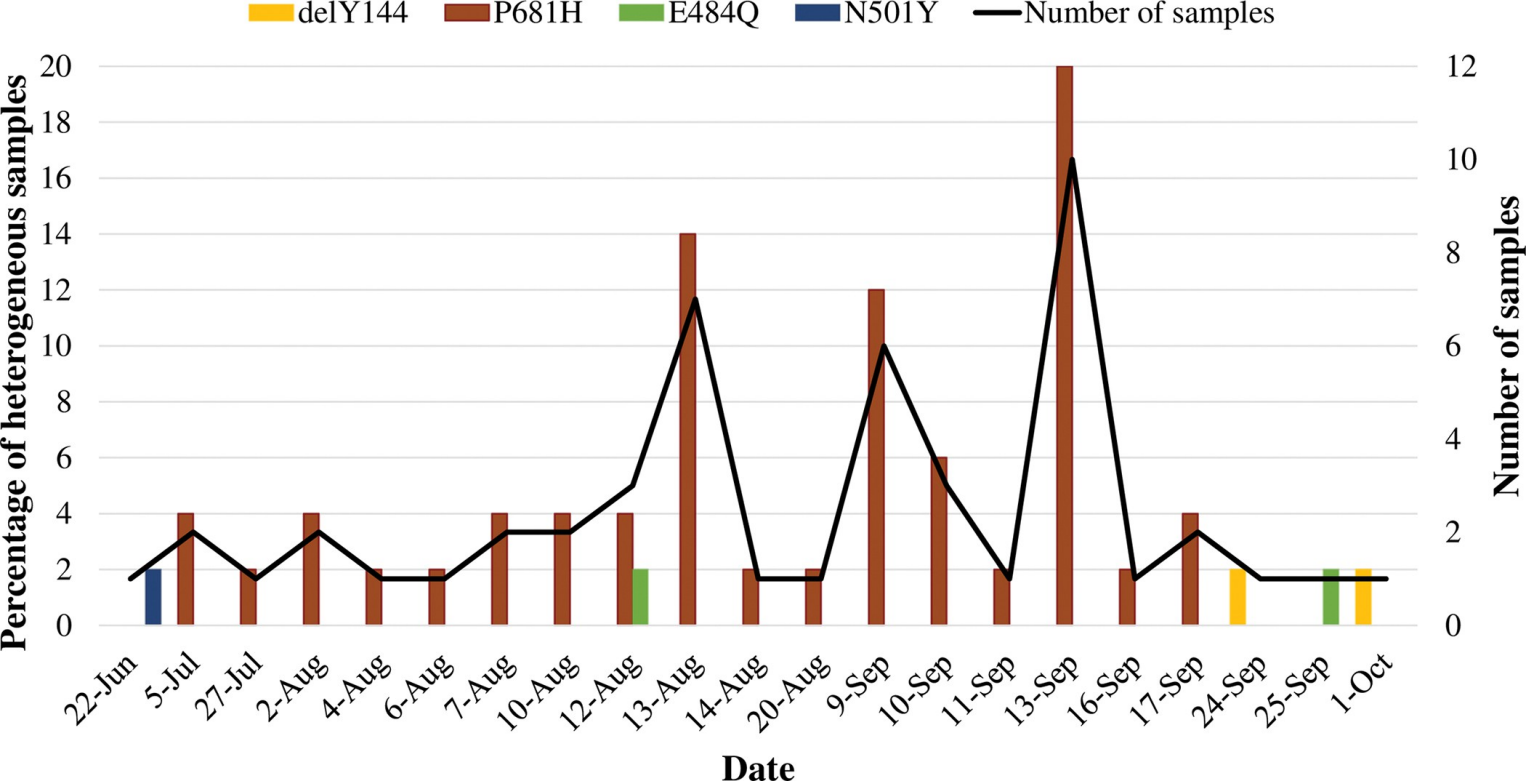
Frequency of the heterozygous SNPs identified from June to October 2021 (N = 50). X-axis represents the date during which the heterozygous SNP was identified. Y-axis: the line graph represents the number of samples that were observed with a particular heterozygous SNP, and the bar graphs represent the total number of samples (N) in which the SNP was observed on each day.

All SNPs tested for were previously reported in SARS-CoV-2 VOCs. Mutations were primarily observed at positions L452 (69.6%; 652/937) [S1E Fig], N501 (5.4%; 51/937) [S1H Fig], D614G (90.1%; 299/332) [S1I Fig], and P681 (82.3%; 771/937) [S1K Fig]. Heterogeneity (mix of wild type alleles at SNP positions indicated by *) was observed for 50/948 (5.3%) cases for only 4 of the 11 SNPs (Table 2; Fig 2). These included the E484Q (2/948; 0.2%), N501Y (1/948; 0.1%), P681H (45/511; 8.8%) and delY144 (2/331; 0.6%). P681R/L452R/P681H* profile of mutations was primarily identified from 17 July to 20 August 2021 and 13 September 2021, whereas the P681R/L452R/P681H*/D614G mutation profile was observed from 9 to 17 September 2021 (Fig 2). Samples with heterogeneous SNPs that were sequenced confirmed heterogeneity at positions 144 (YY144Y, AF = 0.2) and 484 (E484Q, AF = 0.4).

**Table 2:** Summary of SNP combinations with heterogeneous amino acids.

Combination of SNPs (N = 50)	No. of samples n (%)	Heterogeneous positions	Possible VOC/VOI	Sequencing confirmed variant/lineage	Sequencing confirmed heterogeneous amino acid
L452R/delY144*/D614G	1 (2)	delY144	Delta	Poor quality	No^1^
P681R/L452R/delY144*/D614G	1 (2)	delY144	Delta	Delta	YY144Y
P681R/L452R/E484Q/P681H*/D614G	1 (2)	P681H	Delta	Delta	No
P681R/L452R/E484Q*	1 (2)	E484Q	Delta	Delta	E484Q
P681R/L452R/E484Q*/D614G	1 (2)	E484Q	Delta	Delta	E484Q
P681R/L452R/N501Y*	1 (2)	N501Y	Delta	Poor quality	No^1^
P681R/L452R/P681H*	20 (40)	P681H	Delta	Delta	No
P681R/L452R/P681H*/D614G	20 (40)	P681H	Delta	Delta	No
P681R/L452R/P681H*/N501Y	2 (4)	P681H	Delta	Poor quality	No^1^
P681R/L452R/P681H*/del6970	1 (2)	P681H	Alpha/Delta	Delta	No
P681R/P681H*	1 (2)	P681H	Alpha/Delta	Poor quality	No^1^

*heterogeneous amino acids. Poor quality: a VOC or lineage could not be determined due to poor sequence. ^1^Sequence data absent at the amino acid position of interest

We were unable to determine the SNP for 2.0% (21/948) of samples (S1 and S2 Figs) on any of the SNP assays. In addition, undetermined results (negative or no amplification for a SNP) were obtained for 67.5% (345/511) of samples analysed with the P681H SNP assay, 66.3% (479/722) for E484K, 59.0% (196/332) for T20N, 53.2% (505/948) for E484Q, 20.7% (196/948) for L452R, 20.5% (179/873) for del69/70, 17.2% (163/948) for N501Y, 16.0% (152/948) for K417N, 11.2% (27/332) for D614G, 8.1% (77/948) for P681R, and 7.3% (24/331) for delY144. This is despite original PCR diagnostic results with ct values between 13 and 37 (S1 Fig).

### Sequence-based analysis of heterogeneity in S protein

Fifty homogeneous SNPs associated with the primary VOCs detected in South Africa were identified (S3 Table). SNPs identified in Beta included D80A, D215G, K417N, E484K, N501Y, D614G, and A701V with an AF ranging from 0.6–1.0. Individuals infected with Delta had T19R, G142D, delFR157-158, L452R, and T478K (AF ranging from 0.5–1.0) mutations. Forty-one mutations (S3 Table) with an AF of 0.6–1.0, were detected among Omicron-infected individuals. Important mutations at positions R346 and K444 were not detected.

**Fig 3:**
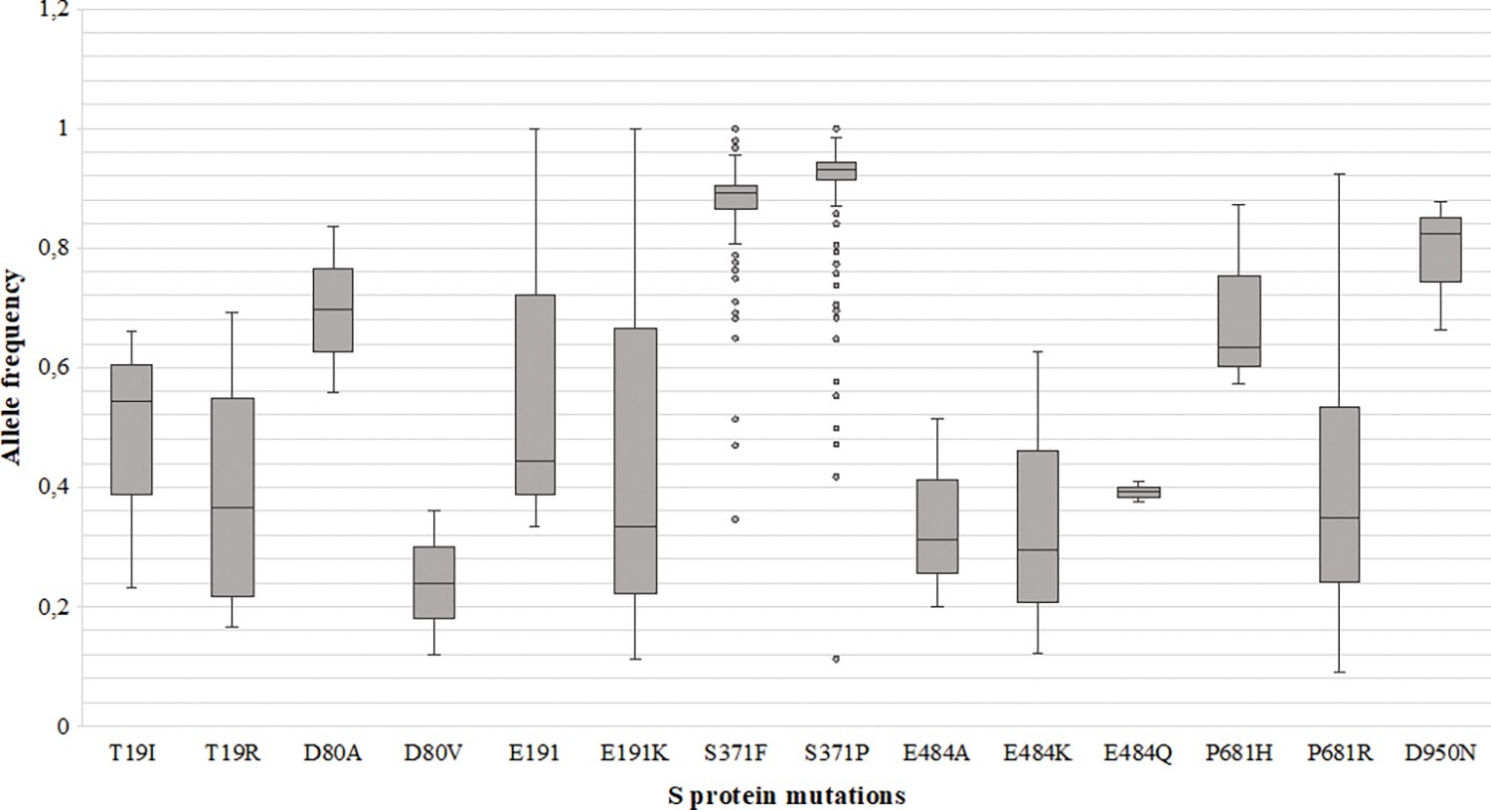
Box and whisker plot showing the mutant allele frequencies (AF) at amino acid positions within the S protein that have more than 1 amino acid change. Circles represent the outliers, line within the box represents the mean AF. Mutations (T19K, P681S D950, D950K, and D950R) that only have a single AF value were excluded from the figure.

Close to 9% (210/2381; 8.8%) of South African SARS-CoV-2 cases sequenced displayed heterogeneity at one or more SNP positions. A total 7 aa positions (19, 80, 191, 371, 484, 681, 950) across the S protein, with AF scores ranging from 0.1 to 1.0, were identified with multiple heterogeneous mutations (Fig 3). At position 19, T19IR mutations (3/210; 1.4%) had an AF of 0.2–0.7 for 19I (C>T, 21618) and 19R (C>G, 21618), whereas T19KR (1/210; 0.5%) had an AF of 0.4 for 19K (C>A, 21618) and 0.5 for 19R. At position 371, the majority (92.9%; 195/210) of cases with heterogeneous mutations had a high AF >0.8–0.9 for 371F (C>T; 22674) and 371P (T>C, 22763) (Fig 3). Lastly, at position 484 among 1.9% (4/210) cases, E484KQ was present with an AF of 0.1 for 484K and 0.4 for 484Q, E484AK had an AF of 0.2 for 484A (A>C, 23013) and 0.7 for 484K (G>A, 23012), and E484AQ had an AF of 0.5 for 484A and 0.4 for 484Q (G>C, 23012).

**Fig 4:**
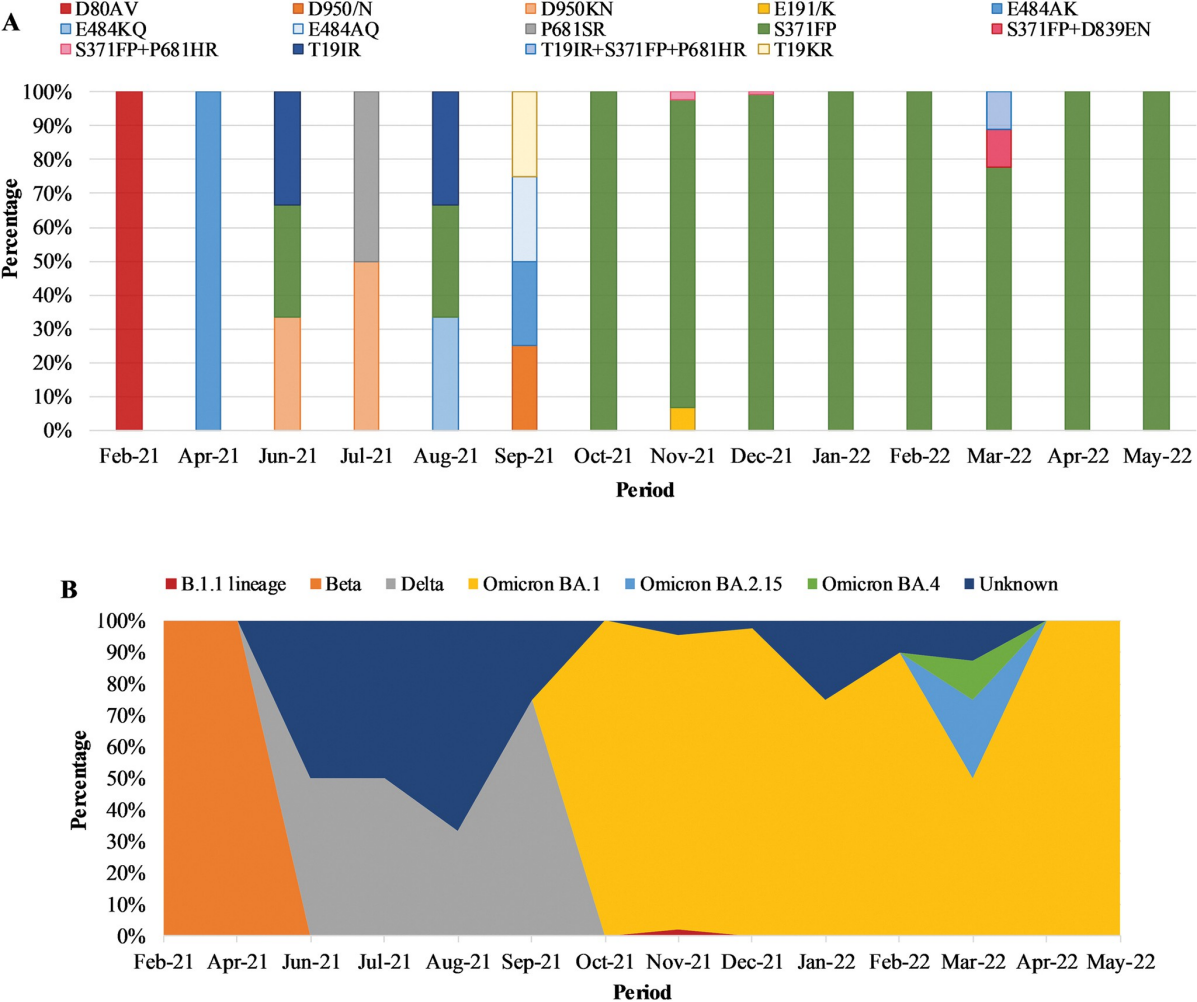
Frequency of the heterozygous mutations (4A) and VOC/lineages (4B) identified from sequence data over time. 4A) X-axis represents the date during which the heterozygous SNP was identified, and the y-axis represents the percentage of samples that were observed with a particular heterozygous SNP. 4B) X-axis represents the period during which the VOC/lineages were identified, and the y-axis represents the percentage of samples with a specific VOC/lineage. Omicron BA.1, BA.1.1, BA.1.10, BA.1.13, BA.1.14, BA.1.17, BA.1.17.2, BA.1.18, BA.1.19 and BA.1.21 were grouped as Omicron BA.1.

**Fig 5:**
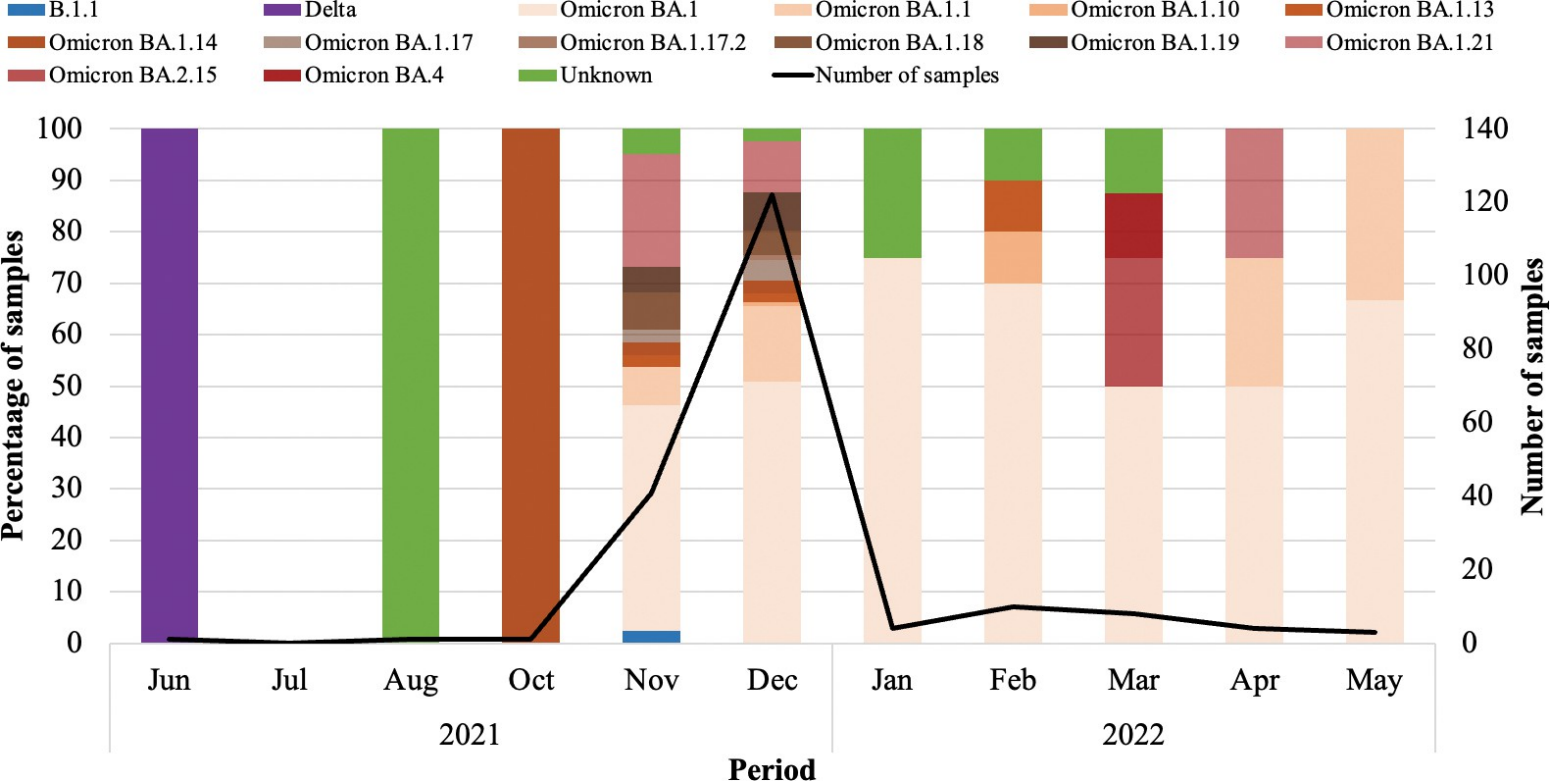
Prevalence of samples with S371FP over time. The black line graph represents the total number of samples with S371FP heterogeneity each month. The bar graphs represent the SARS-CoV-2 VOCs and lineages observed with the S371FP heterogeneous mutation from 2021 to 2022.

Of the 3 samples with T19IR heterogeneous mutations, each sample was classified as Delta (June 2021), Omicron BA.21.5 (March 2022), and unassigned (August 2022) (Fig 4). The sample with T19KR was identified in October 2021 but could not be classified (unassigned) due to lower sequence quality at other regions of the genome. S371FP heterogeneous mutations were identified from June 2021 through to May 2022 of which 1/195 (0.5%) was classified as Delta. The S371FP heterogeneous mutation rapidly increased from the Delta (June to August 2021) to the Omicron waves and dominated during the Omicron BA.1/BA.2 wave from November 2021 (41/195; 21.0%) to December 2021 (122/195; 62.6%) (Fig 5). From January 2022 to May 2022 the S371FP mutation was observed but at lower frequencies from 2.1% (4/195) to 5.1% (10/195) from the Omicron BA.1/BA.2 wave to the Omicron BA.4/BA.5 wave. The majority of S371FP was identified among 92.8% (181/195) of cases classified as Omicron BA.1 lineage strains of which 50.3% (98/195) were BA.1, 11.8% (23/195) were BA.1.1, 11.3% (22/195) were BA.1.21, and the remaining 19.5% (38/195) were BA.1.10, BA.1.13, BA.1.14, BA.1.17, BA.1.17.2, BA.1.18, and BA.1.19. S371FP was also detected in 1.0% (2/195) Omicron BA.2.15, 0.5% (1/195) Omicron BA.4, and 0.5% (1/195) B.1.1 lineages. The lineage or VOC could not be determined for 4.6% (9/195) of the cases due to low sequence data quality in some regions of the S gene (Fig 4). The majority (146/195; 74.9%) of cases with the S371FP were from individuals that sought CST, while the remaining cases (25.2%) were from deceased individuals (1/195; 0.5%), HCW (4/195; 2.1%), in-patients (24/195; 12.3%) or out-patients (20/195; 10.3%). Individuals aged 5–14 (43/195; 22.1%), 15–24 (48/195; 24.6%), and 25–44 (58/195; 29.7%) were the predominant age groups with S371FP (S1 and S2 Tables). Three percent (5/195) of out-patients visited ARV clinics. Two cases with multiple heterogeneous mutations which included S371FP/P681HR were classified as B.1.1 and Omicron BA.1 lineages; detected in November 2021 and December 2021 from CST, respectively. Whereas T19IR/S371FP/P681HR and S371FP/D389EN mutation profiles were identified in March 2022 among sequences from 2 cases classified respectively as Omicron BA.2.15 and Omicron BA.4 from CST (Fig 4). E484KQ (1/4; 25%) was observed in an out-patient from an ARV clinic in August 2022 and classified as Delta (Fig 4). E484AK (2/4; 50%) and E484AQ (1/4; 25%) were identified among individuals that sought CST. E484AK was observed among samples classified as Beta and Delta in April 2021 and October 2021, respectively. While E484AQ (1/4; 25%) was identified in October 2021, and classified as Delta.

### Mutations at dominant heterogeneous SNPs positions compared to global data

**Fig 6:**
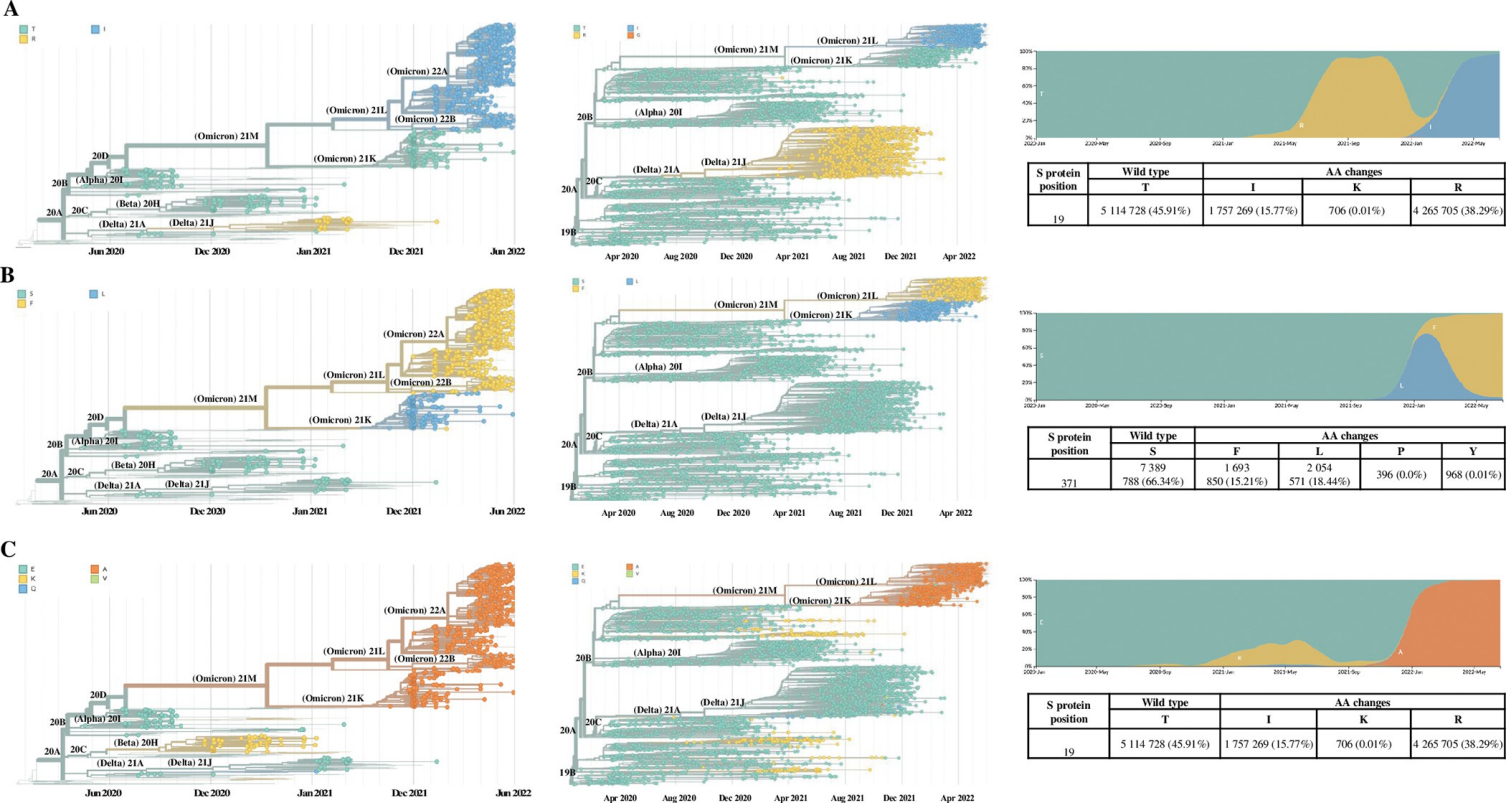
Frequency of mutations occurring at aa positions 19, 371, and 484 in the S protein. Custom NextStrain time-tree (left) displays the sequence data from this study from June 2020 to May 2022, with specific aa changes at the heterogeneous positions. The phylogenetic tree (middle) represents the global SARS-CoV-2 sequence data from January 2020 to May 2022, with specific aa changes at the heterogeneous positions (https://nextstrain.org/ncov/gisaid/global/all-time, accessed 22 June 2022). Clades displayed in phylogenetic trees represent the following SARS-CoV-2 pangolin lineages or VOCs (https://covariants.org/variants): 20A and 20C include B.1 lineages, 20B includes B.1.1 and its sub-lineages; 20D includes B.1.1.1 lineage, Delta 21A, and Delta 21J includes B.1.617.2 and AY lineages; Alpha 20I includes B.1.1.7; Omicron 21M includes B.1.1.529 lineage; Omicron 21K includes BA.1 lineage; Omicron 21L includes BA.2 lineages and branches out to Omicron 22A (BA.4) and Omicron 22B (BA.5). Top right graph shows the frequency of the aa changes over time (https://nextstrain.org/ncov/gisaid/global, accessed 22 June 2022). Tables show the frequency of wild-type aa and mutations present globally from GISAID (https://www.gisaid.org/epiflu-applications/covsurver-mutations-app/, accessed 22 June 2022).

The consensus sequence data was used to compare our data to global data. Globally, from January 2020 through to May 2022, multiple aa changes were seen at positions 19, 371, and 484 among 11 140 058 SARS-CoV-2 genome sequences uploaded on GISAID (accessed 22 June 2022). At aa position 19, the T19I mutation was detected in 15.8% of global sequences (January 2022 through May 2022) in Omicron BA.2 (21L clade) and T19R was identified among 38.3% of Delta lineages from March 2021 through March 2022, similarly identified in our study sequences (Fig 6A). At position 371, the S371F mutation was globally dominant (15.21%) and identified from December 2021 to May 2022 among Omicron BA.2 lineages, while our study identified S371F primarily among Omicron BA.4 (22A clade) which clustered out from Omicron BA.2 from June 2021 to May 2022. S371P is not yet reflected since it was only observed in June 2022 in 396 global genomes and was not observed from our data due to S371F being the dominant mutation from the consensus sequences. S371L was identified among our consensus sequences but was not observed among the heterogeneous mutations. At position 484, E484A was identified in the majority (34.1%) of global sequences from November 2021 to May 2022 and classified as Omicron BA.1 and BA.2 lineages, similar to our findings but also identified among Omicron BA. 4 and BA.5 (22B clade). Whereas E484K was identified in 2.3% of global sequences belonging to all lineages except Omicron lineages. E484Q was observed among 0.2% of sequences from Beta and Delta lineages detected from May 2021 to December 2022.

## Discussion

Intra-host SARS-CoV-2 heterogeneity results in greater viral diversity and viral evolutionary advantage to enhance virus-host adaptation, immune evasion, replication, transmission, and pathogenicity [10, 17, 18]. Diversity studies identified high variability of iSNVs in several regions of the genome such as the ORF and N genes of SARS-CoV-2, with a high frequency of allelic variations, especially within ORF8 and negative selective pressure towards the S gene. The mutation profiles of intra-host quasispecies were also shown to change from the onset of symptoms which could contribute to viral evolution occurring during the course of infection [10, 17, 18]. Our study assessed the intra-host diversity based on the heterogeneity of SNPs across the S gene, not previously described in South Africa, to postulate the impact on host immune responses.

In contrast to earlier studies, our study period from June 2020 to May 2022 encompassed five SARS-COV-2/COVID-19 infection waves in South Africa. The 4^th^ wave, driven by the Omicron BA.1 and BA.2 lineages, had the highest detection rate peaking at 58%, while the lowest peak detection rate (24%) was observed during the Omicron BA.4/BA.5 driven wave of infections [19, 20]. Although the majority of samples included in this study were from the Gauteng province, the overall distribution of SARS-CoV-2 lineages and VOCs was similar in other Provinces of South Africa [19, 21]. Individuals that seek CST are usually asymptomatic or present with mild symptoms as a result of a causative agent other than SARS-CoV-2, therefore a drop in the detection rate was observed. It has been reported that individuals infected with Omicron have reduced severity of infection compared to Delta as well as immunity resulting from natural infection and vaccination within the population [22, 23]. Of the cases that tested positive for SARS-CoV-2 in this study, both males and females were predominantly from the 25–44 year age group and CST, with the majority belonging to females (59%).

SARS-CoV-2 S protein SNP analysis identified mixed alleles indicative of heterogeneous populations or quasispecies among 5.3% of cases (which define heterogeneous populations or quasispecies). Heterogeneous genotypes were detected mainly at one or more of the S protein aa positions: 144, 484, 501 and 681, where P681H was the most frequently observed. Overall, sequencing-based variant calling analysis identified 8.8% heterogeneous infections among our study population are heterogeneous infections, which included 3 samples analysed by SNP PCR. These results contrast with a study conducted in 2020 in Australia which reported quasispecies diversity in S protein at a frequency of 12% (13/107), although their sample size was much smaller [24]. We were unable to ascertain if study participants were infected with mixed virus populations, had multiple exposures/infections closely timed, or if the mixed populations observed were due to *de novo* evolution in the infected host during the course of illness.

The lower frequency of heterogeneity reported here for SNP-based approaches could be ascribed to the lower sensitivity of SNP assays. In some cases, more than 50% of samples could not be analysed for SNPs despite acceptable cycle threshold values obtained with the diagnostic tests. Of the heterogeneous SNPs identified, only aa position 144 was truly heterogeneous from sequencing. P681H was the dominant heterogeneous SNP observed during the tail-end of the 3^rd^ COVID-19 wave, but sequencing confirmed that this position had the wild type or mutant at position 681, but not both. Amino acid mutations that usually occur adjacent to each other in the SARS-CoV-2 genome, including P681H and P681R, make genotyping analysis challenging since the probes in the assay for one mutation failed to bind to viral sequence with the adjacent mutations [25]. Mutations at this position appear as mixed (wild type/mutant) and moved away from wild type/wild type or mutant/mutant assignment as a result of weak amplification due to non-specific probe activity was evident. The latter study tested K417N, L452R, E484K, N501Y, and P681R; and only identified heterozygosity for E484K (1/42; 0.6%) and P681R (8/42; 19.0%) from the SNP assays, not observed in our findings. Another study that tested 13 S protein TaqMan SNP assays, excluding P681H but including additional T20N, del242, and T478K; showed a 100% concordance when comparing the SNP assay to sequencing data [26]. However, only 9/150 samples were confirmed using sequencing, and comparisons were based on lineage/VOC calling rather than the frequency of wild type and/or mutation.

Of the samples sequenced, heterogeneity was primarily identified at aa positions 19 (T19IR and T19KR), 371 (S371FP), and 484 (E484AK, E484AQ, E484KQ), similar to the SNP PCR which identified heterogeneity of the S protein at the aforementioned aa positions, before the 3^rd^ wave through to the 5^th^ wave of infections in South Africa. We identified T19IR among individuals that tested during CST and had Omicron BA.2.15 infection, while the lineage information for those with T19KR mutations was unknown due to missing sequence data. Amino acid position 19 is present in the N-terminal domain (NTD) of the S gene, and mutations at this position previously identified in Delta and Omicron BA.2 [27, 28]. The site I (“supersite”) on the NTD is usually the target for NTD-specific neutralising antibodies. However, the T19R mutation disrupts S protein conformation, which reduces virus neutralisation by antibodies [29]. When comparing the mutations observed in our study to the global context, high frequencies of T19I were observed among Omicron BA.2 lineages and T19R among Delta. T19K was observed at 0.01% and in Alpha VOC that circulated at low frequencies in South Africa [6].

Our data showed that S371FP was identified among Omicron BA.1, BA.2.15, and BA.4 lineages. S371F was observed at much higher frequencies compared to S371P, both of which were identified in the Omicron BA.1 and BA.2. S371L was not associated with heterogeneous infections in our study but was identified as the dominant mutation globally among Omicron BA.1 lineage strains. Mutations at position S371 led to a reduced recognition of the receptor-binding domain (RBD) antibodies [27, 28]. Similar to our study, S371P, and S371F were reported among Omicron BA.2 lineages in Europe and Italy, and in BA.3 lineage which was sporadic in South Africa [20, 28]. Despite the fact that the region around S371 to F541 does not have any N-linked glycosylation sites, aa changes at position 371 still decrease neutralising antibody recognition [30]. S371P is recognised as a mutation that results in virus escape from neutralisation by antibodies [31], while S371F alters the conformation of the RBD which results in neutralising antibody escape [32]. The most frequently observed heterogeneity was among individuals in age groups 5–14 (22.0%), 15–24 (23.3%), and 25–44 (32.9%). Although limited information on HIV-1 status among study participants including out-patients that visited ARV clinics and had heterogeneous mutations, recent data showed that the HIV-1 prevalence among South Africans aged 15 to 49 years remains high at 19.5% [33]. A shedding study done in South Africa showed that persons living with HIV/AIDS with low CD4 counts (<200cells/μl) had significantly longer periods of SARS-CoV-2 shedding, ranging between 7 and 43 days, during the 2020 pandemic period in South Africa [34]. A longer period of virus shedding provides an opportunity for the virus to evolve in its efforts to evade the SARS-CoV-2 specific immune responses [35–37]. Omicron BA.1 was initially detected in HIV-infected patients in Botswana, South Africa, suggesting that individuals with underlying medical conditions such as HIV infection are at a higher risk of SARS-CoV-2 infection and severe disease as well as a possible predisposition for evolving SARS-CoV-2 mutants [38].

We identified E484AK, E484AQ, and E484KQ heterogeneity at very low frequencies among Delta and Beta variants from CST persons and an ARV clinic patient. Unlike our study, E484 mutations were not previously detected in Delta but we could speculate that the virus underwent adaptive changes since E484AK/AQ/KQ were identified among cases just after the Delta (3^rd^) wave, leading towards the Omicron wave. Globally, E484A was observed at the highest frequency and was primarily observed in Omicron BA.1 and BA.2 lineages. E484K was less frequent but appeared in all lineages over time, with the exception of Omicron. Previously studies reported E484A in Omicron BA.1, BA.2, BA.3, BA.4, and BA.5 lineages; E484K was identified in C.1.2, Beta, Alpha, Gamma, Eta, Lota, and Mu; and E484Q in Kappa and B.1.595 lineages [20, 39, 40]. The E484A, E484K, and E484Q mutations reduce sensitivity to vaccine-induced antibodies through immune escape from class 2 neutralising antibodies, with increased transmissibility due to higher affinity for the host receptor [39, 41–43]. Of note is that E484 was the only site for which vaccine data was available compared to T19 and S371. The E484Q mutation is associated with reduced resistance to neutralisation by post-vaccination sera, and two doses of mRNA vaccines provide protection against E484Q variants in adults [39, 40]. E484K has shown evidence of neutralisation by the monoclonal antibodies bamlanivumab and etesevimab as well as convalescent sera, but reduced neutralising antibody activity was observed among Pfizer-BioNTech and Moderna vaccine recipients [40].

Other studies reported significant non-synonymous SNVs in the S gene reported early in the pandemic during 2020, which include mutations at nucleotide position G22899T, which translates to amino acid mutation G446V in the RBD, and G24557T which translates to a G999C mutation in the heptad repeat region associated with membrane fusion during virus entry [24]. Similarly, another study identified nucleotide substitutions at positions 22899 (translates to G446V), 21575 (L5F associated with increased infectivity in-vitro), and 24198 (A879V with reported reduced sensitivity to neutralisation by convalescent sera in-vitro) which are present in the RBD of the S protein [26]. However, these mutations were not observed in our study. Several mutations in the spike protein are responsible for neutralising antibody escape. Group A-D antibodies comprise the strongest antibodies which disrupt binding to the ACE-2 receptor and escape neutralisation from Omicron lineages [44]. Omicron lineages are associated with escaping neutralisation from various antibody groups. Group D antibodies were affected by mutations at positions 440, 444, 446, and 448 found in the Omicron lineages [44]. While group E neutralising antibodies are sensitive to mutations at positions including 339, 345, and 346, with the frequency of R346K mutations rapidly increasing with emerging Omicron lineages. The primary mutations known to evade neutralisation by antibodies in Beta and Delta include the mutations at positions 484 and 478 [45, 46]. Of the described mutations, we identified changes at aa homogeneous positions 339, 478, and 484 where 484 was also heterogeneous.

Heterogeneity among our study samples was predominantly observed from June 2021 through to May 2022 covering the Delta to Omicron dominant waves. It was also evident among individuals that sought CST and were expected to be asymptomatic or present with mild influenza-like illness symptoms. However, in this study the majority of cases were from CST for which clinical data were not collected; therefore, we could not conclude that heterogeneous SARS-CoV-2 infections were related to the severity of the disease. Another contributing factor to the observed heterogeneity may be immune selective pressures and adaptation when virus transmission occurs between persons with different HLA backgrounds [24].

The first limitation of the study was the performance of the T20N, P681H, E484K, and E484Q genotyping assays. The cycle-threshold (Ct) values, of samples from diagnostic SARS-CoV-2 testing, were compared to the genotyping assay results to determine the success of each assay. We genotyped most samples that tested positive for SARS-CoV-2 with Ct-values ranging from 13 to 37 from diagnostic PCR assays. However, it is important to note that only two of these assays relate to heterogeneous SNP positions identified by variant calling analysis. We, therefore, concluded that the reduced sensitivity of some Thermo Fisher SNP assays to distinguish wild-type from mutant might be due to the negative impact of other mutations surrounding the SNP of interest [25]. Secondly, incomplete or low-quality S gene sequence coverage was generated from whole genome based NGS due to primer mismatches as a result of novel variants emerging and becoming dominant in each wave of infection. We acknowledge that this problem could have been overcome by updating amplification primers or spiking the primers, but we did not re-sequence samples due to cost. Thirdly, our study is not a longitudinal study but rather describes the heterogeneity across the entire population, looking at the diversity and dominance of heterogeneous mutations within lineages and VOCs over time. Fourth, samples included in this study were primarily from the Gauteng Province, although the lineages and VOCs circulated similarly to that observed in the other Provinces of South Africa, we could not assume that similar heterogeneity would occur throughout the country. A fifth limitation was that we did not have the clinical data at the time of the study for individuals that were tested as part of community testing and from out-patients and encompassed most of the cohort. Lastly, we did not perform antibody neutralisation assays to determine the significance of immune adaptations during the different waves of infection among individuals infected with the heterogenous mutations detected.

## Conclusion

Unlike other intra-host diversity studies that focused on mutations of the SARS-CoV-2 genome, our study analysed the S protein aa changes as it is the primary target for neutralising antibodies and this focus for viral immune evasion. Substitutions at aa positions 19, 371, and 484 have previously been shown to reduce virus neutralisation by antibodies. However, we are uncertain if the impact is less or greater among persons infected with SARS-CoV-2 that display heterogeneity in the S protein. Low-frequency variants at heterogeneous aa positions across the S protein provide opportunities for the emergence of SARS-CoV-2 variants that can overcome or outcompete when conditions favour survival and transmission. Our findings show that heterogeneous mutations may be a result of intra-host adaptation and may drive the evolution of the S protein which could contribute to immune evasion. This may contribute to the ongoing emergence of new variants associated with continued outbreaks globally. Accounting for diversity in vaccine design and universal vaccine design strategies may be necessary to ensure effective vaccines to protect against all variants of SARS-CoV-2.

## Supporting information

S1 Fig Distribution of Ct-values for each SARS-CoV-2 mutations tested. (TIF)

S2 Fig Allelic discrimination plots of mutants, wild type and heterogeneous SNPs (mutant/wild type). (TIF)

S1 Table Patient status for cases where heterogeneity in SARS-CoV-2 S protein was observed. (TIF)

S2 Table Age groups for cases for which heterogeneity in S was identified. (TIF)

S3 Table Allele frequency and prevalence of homogeneous mutations identified among all individuals in the population. (TIF)

S4 Table Accession numbers for fastq data for heterogeneous samples. (TIF)
