# Supplementary figures and images for: SARS-CoV-2 spike protein diversity at an intra-host level, among SARS-CoV-2 infected individuals in South Africa, 2020 to 2022
Source: PLoS One. 2023 May 30;18(5):e0286373. doi: 10.1371/journal.pone.0286373 (PMC10228762; doi:10.1371/journal.pone.0286373)

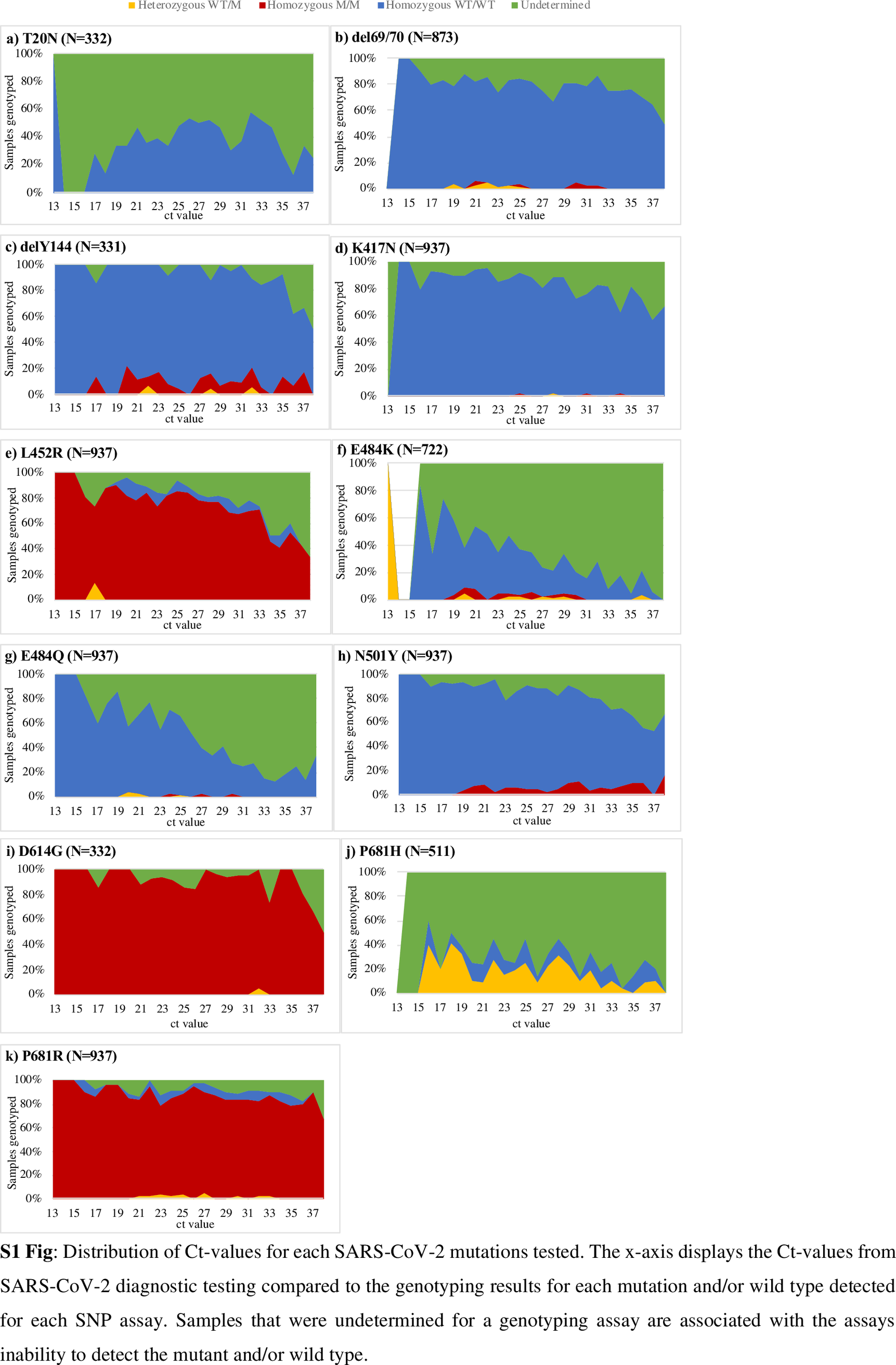

Supplement: S1 Fig — (TIF) [file pone.0286373.s001.tif]

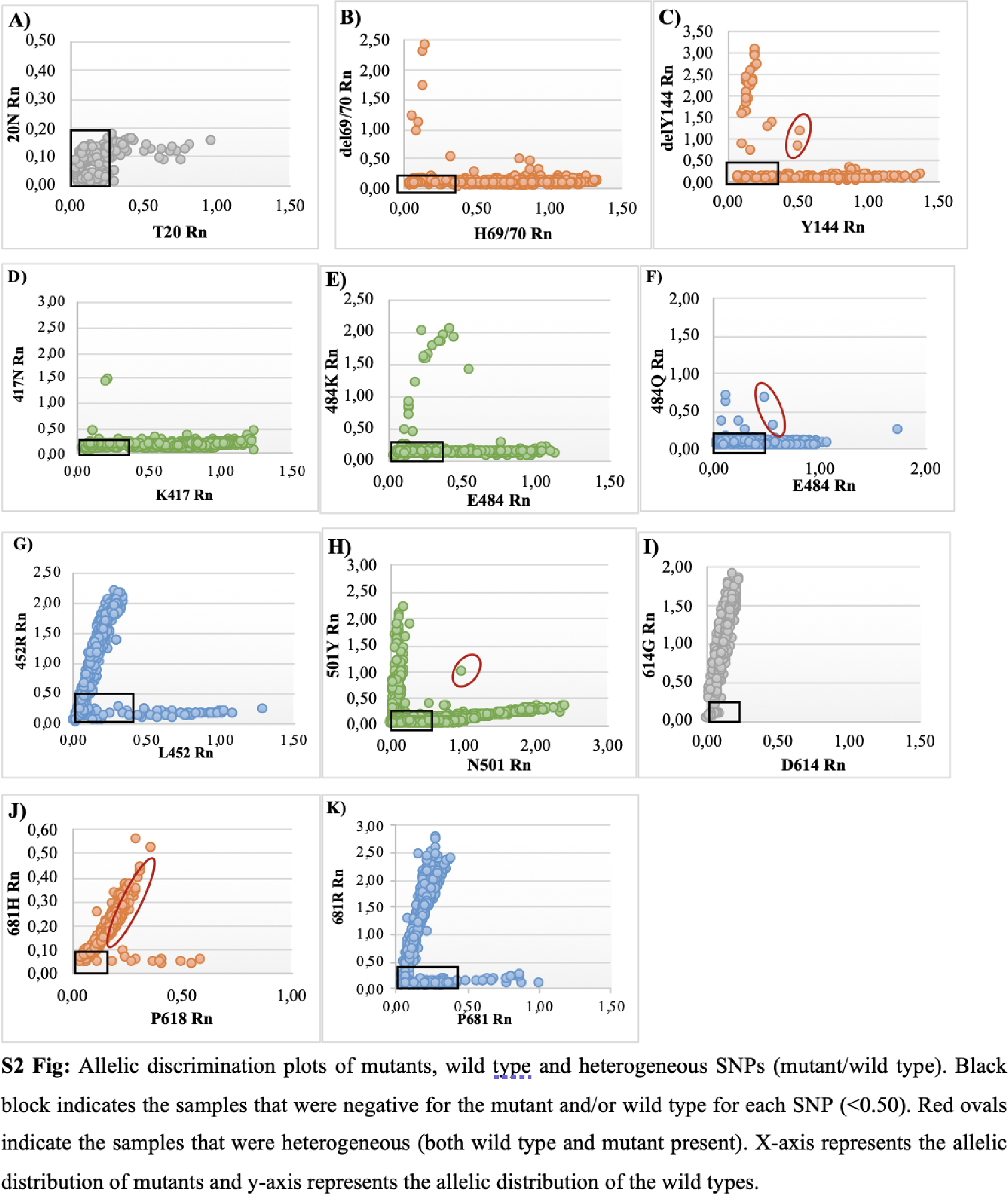

Supplement: S2 Fig — (TIF) [file pone.0286373.s002.tif]

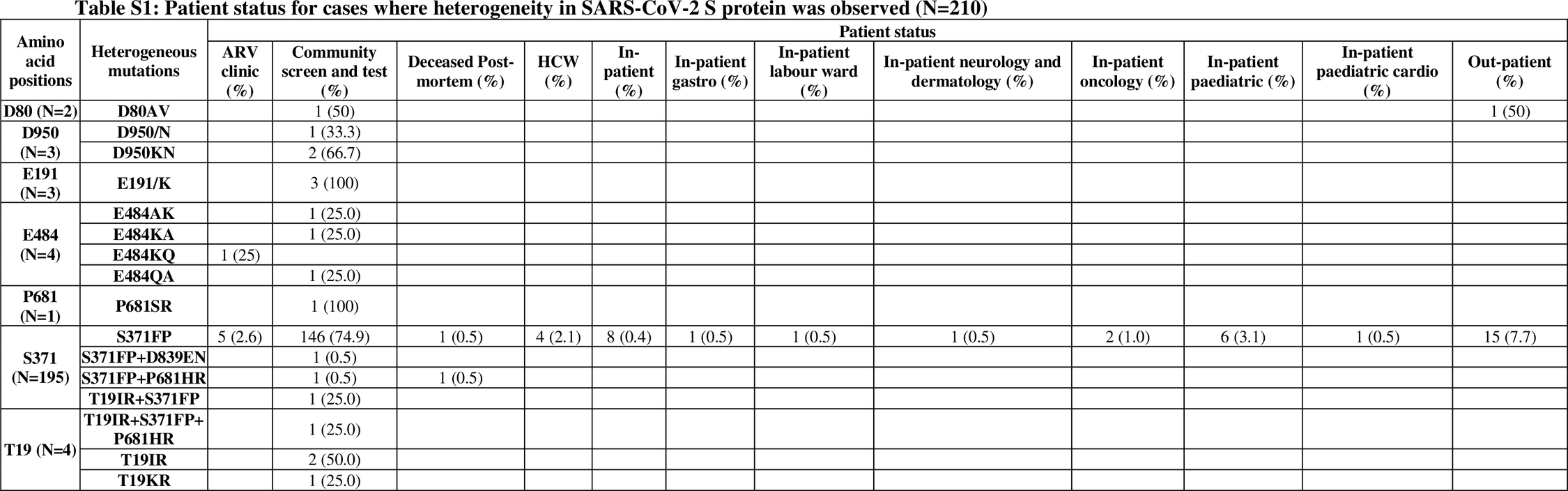

Supplement: S1 Table — (TIF) [file pone.0286373.s003.tif]

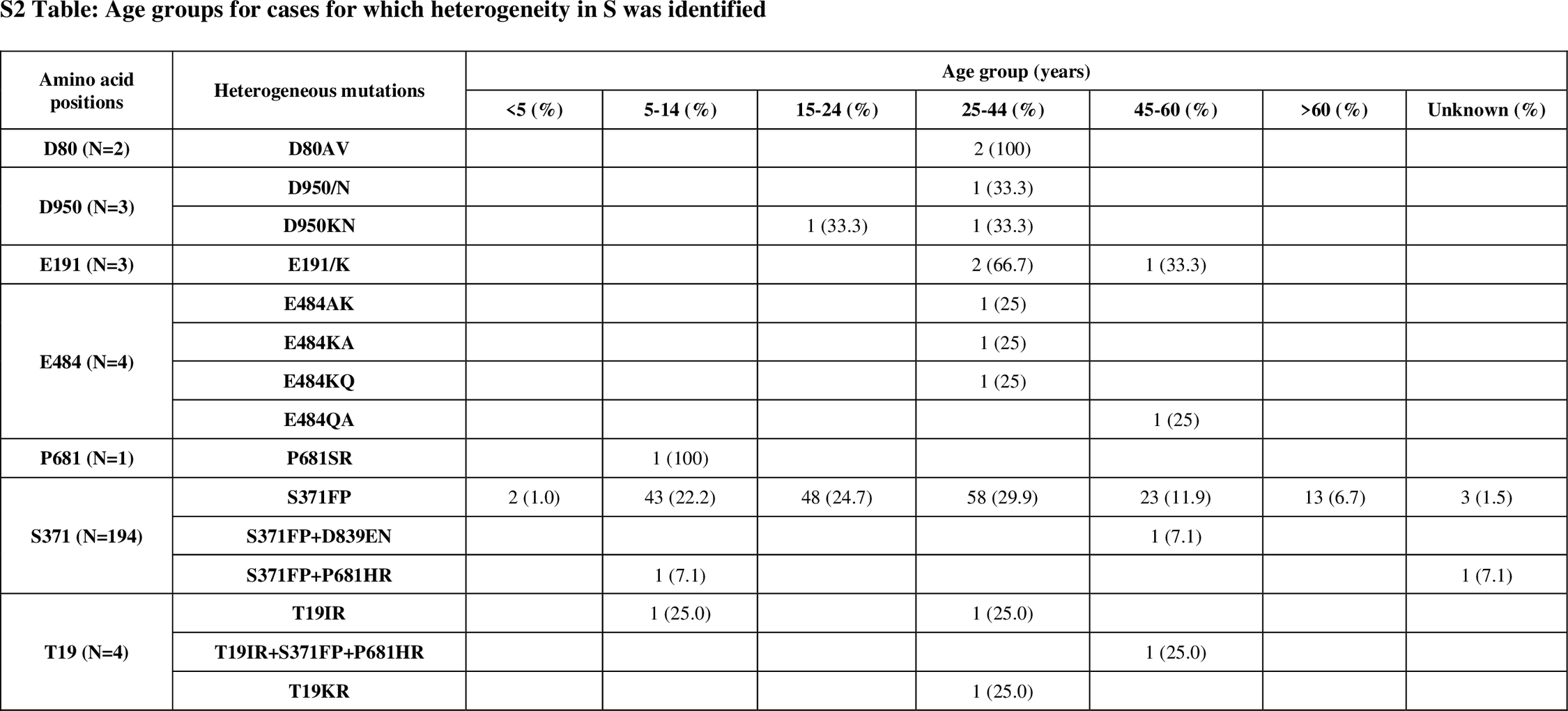

Supplement: S2 Table — (TIF) [file pone.0286373.s004.tif]

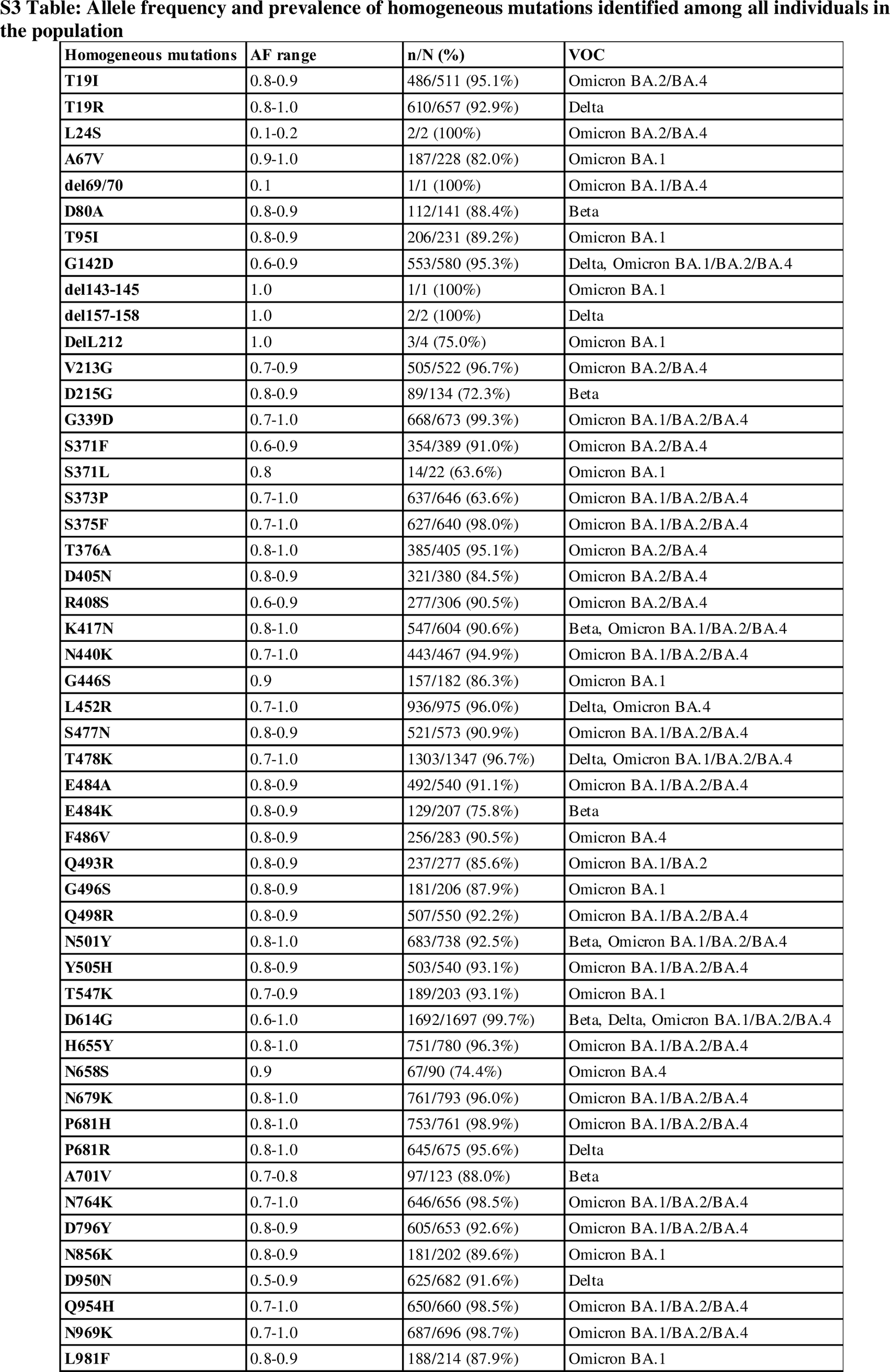

Supplement: S3 Table — (TIF) [file pone.0286373.s005.tif]

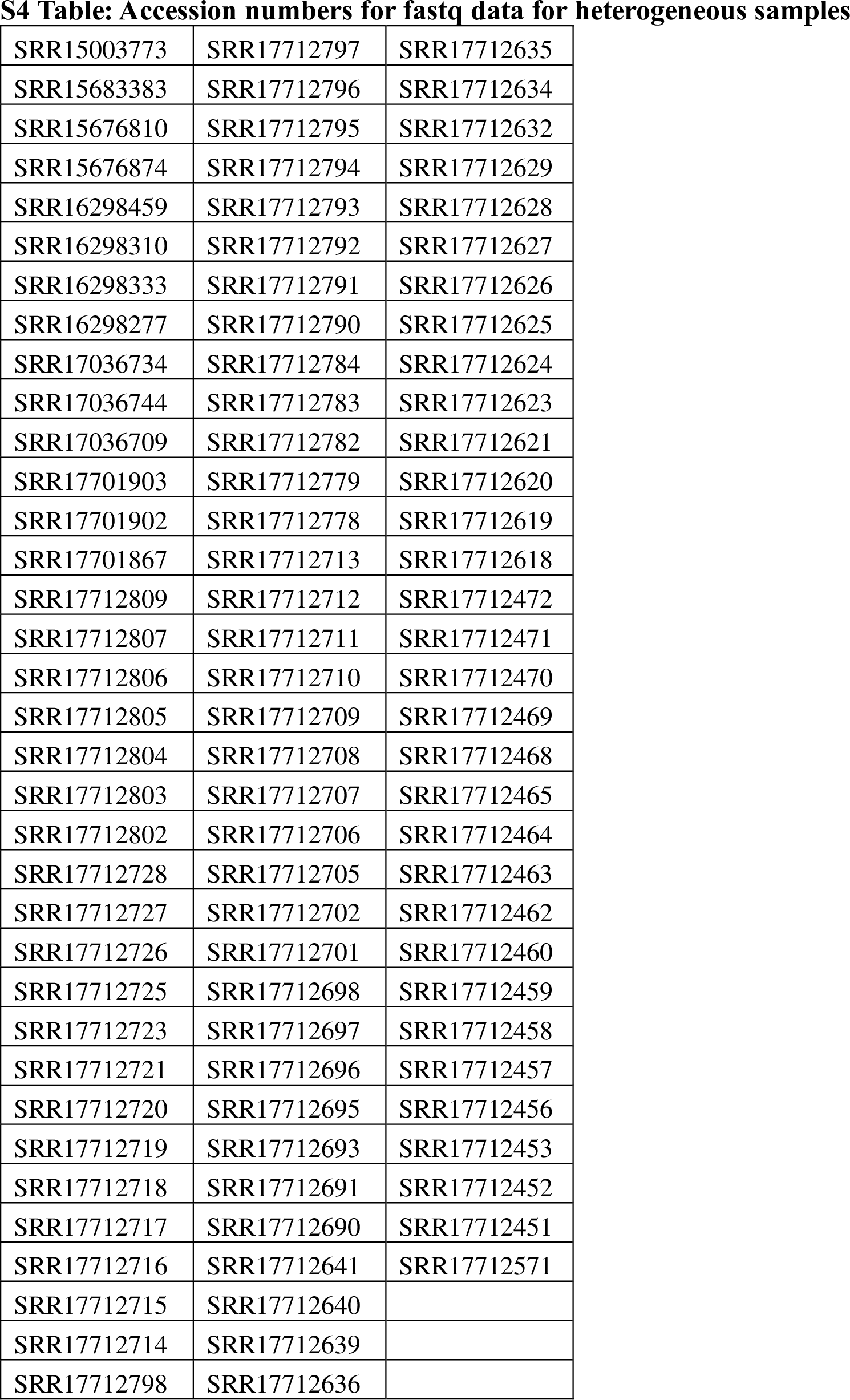

Supplement: S4 Table — (TIF) [file pone.0286373.s006.tif]
